# Exposure to occupational noise: otoacoustic emissions test alterations

**DOI:** 10.1016/S1808-8694(15)30969-1

**Published:** 2015-10-19

**Authors:** Frederico Prudente Marques, Everardo Andrade da Costa

**Affiliations:** a MS in Public Health, Otorhinolaryngologist at the CEMEP Hospital; b PhD in Collective Health - UNICAMP – MS in Communications Disorders - PUC-SP, Otorhinolaryngologist, Professor from the Department of Social and Preventive Medicine - UNICAMP. School of Public Health – University of São Paulo

**Keywords:** otoacoustics emissions, hearing loss, occupational noise

## Abstract

Exposure to occupational noise may cause injuries to the inner ear, and the distortion product otoacoustic emissions (DPOAE) may identify initial auditory alterations, thus assisting NIHL early diagnosis.

**Aim:**

The goal of this study was to evaluate DPOAE as a method to diagnose early physiopathological alterations caused by occupational noise exposure.

**Study Design:**

Transversal.

**Methods:**

74 workers of the University of São Paulo, in the capital city of the State, participated in this investigation. They were divided in two age-matched groups and with tonal audiometric values within the acceptable limits: 37 were exposed to occupational noise and 37 were not exposed.

**Results:**

Risk estimates (Odds Ratio) of absent DPOAE was 12 fold higher for the group exposed to occupational noise (CI 95% 3.1 - 45.9), in the frequencies of 3. 4 and 6 kHz.

**Conclusions:**

DPOAE may be useful in the identification of physiopathological hearing alterations caused by exposure to occupational noise, even in individuals with tonal audiometric responses within acceptable limits.

## INTRODUCTION

Exposure to noise or high sound pressure levels is the main, and preventable, cause of sensorineural hearing loss in adults^1^. A constant concern of public health management has been such exposure and its consequence for hearing. Therefore, a growing number of studies have cropped up in attempts to better understand and limit the occurrence of Noise Induced Hearing Loss caused by Occupational Noise (ONIHL)^2^, ^3^, ^4^.

Noise exposure is a risk to the worker's health, which may disturb work, rest, sleep and even the communication between human beings. ONIHL is an insidious disease, growing along the years, having a direct relationship with intensity, time of exposure and individual susceptibility to noise^2^.

Threshold tonal audiometry is the universally accepted method employed in order to diagnose ONIHL, however, according to Costa^5^, this is not the best means to asses noise induced disorders, because it tests the individual's capacity to hear a pure tone and in different situations of his daily activities. Glorig^4^ reports that initial lesions to the hearing apparatus are not detected by audiometry - they are only diagnosed after the damage has become irreversible.

The possibility of using alternative methods to detect hearing alterations caused by exposure to high sound pressure levels is extremely important, since the very interpretation of audiometric test results may direct influence the worker's professional life. Besides, it is important that the health care professional detect early on the first signs of hearing involvement, and because he/she is not a specialist, they need a simple and efficient method.

Distortion Product Otoacoustic Emissions (DPOAE) has been considered because it reveals early alterations brought about by noise exposure^6^, ^7^, and it may aid the physician in assessing the workers exposed to this risk.

Distortion Product Otoacoustic Emissions are evoked by a bitonal sound stimulus. During the exam, two pure tones trigger all the active process of sound frequency discrimination in the hearing system. Distortion products are obtained as the result of vibratory energy generated at the cochlea, which may be measured by means of a microphone coupled to the ear of the individual being tested^8^.

Fiorini^9^ says that in epidemiological surveillance of hearing alterations caused by noise exposure, the otoacoustic emissions test allows us to obtain important clinical information that completes audiometric data.

In the present study, we used DPOAE as a test that is able to identify initial hearing impairment, thus contributing to the diagnosis of ONIHL and to preclude the development of these hearing losses.

The goal of the present study is to investigate if indeed DPOAE are able to identify early hearing impairments related to occupational noise exposure, even when tonal audiometry is normal.

## MATERIALS AND METHODS

A transversal study was carried out, including two groups of individuals, those that have been exposed and those who have not been exposed to occupational noise, with tonal thresholds within acceptable limits, assessed by means of distortion product otoacoustic emissions recordings.

Employees of the University of São Paulo Campus at the Capital participated on the study.

We collected secondary data on sound pressure levels measurements carried out on different units of the University of São Paulo Campus at the Capital: Institute of Physics, Administration of the University Campus, Polytechnic School, Social Communications Coordination, and Residential Area of the University of São Paulo, Oceanographic Institute, Electronic Engineering Institute, Communications and Arts School. Such analysis allowed us to group the places where there was environmental noise above the tolerance levels established by law^10^ and, consequently, risk for the workers’ hearing – environmental noise that determined exposure during the work shift of 8 hours, at an intensity above 85 dB (A).

We analyzed the tests carried out between April 2001 and March 2002 (12 months period), a total of 263, in order to find the tests of those employees who had been working for at least one year in areas that presented sound pressure noises above the tolerance levels defined by the Brazilian Legislation.

We also selected, among exposed employees, those with tonal audiometry tests within acceptable limits, according to Ordinance # 19 of the Ministry of Employment and work^11^, in other words, up to 25 dB HL in all the frequencies, from 250 to 8.000 Hz.

Matching the results from tonal audiometry and noise exposure at the working environment, we found 50 workers. From this group, a total of 13 workers could not undergo the otoemissions test: 6 refused to participate in the research and 7 no longer were University employees. We then formed Group I, 37 workers.

As to gender, initially there were females eligible to participate in Group I; all of them were telephone operators. It was not technically possible to determine the sound pressure levels to which these individuals were exposed. We did not find any conclusive scientific paper related to this theme. Thus, the groups of patients exposed and not exposed to noise were formed by males only.

The reference group (GROUP II), used to compare the Otoacoustic Emissions Tests with Group I, counted on workers from the same institution who had not been exposed to occupational noise in their current or previous occupations.

Knowing the composition of the group of exposed individuals made it possible to establish age limits and their separation by age ranges, and to keep a similar proportion of individuals in the same age range for each group.

Among all the people selected and contacted to participate in this group, 6 individuals refused to participate and 5 had altered audiometric exams. At each refusal or audiometric failure, another individual was randomly chosen for replacement, and the age range was previously selected in order to continue the pairing process. Thus, 37 individuals made up Group II.

The tests were carried out after at least 14 hours of acoustic rest for those individuals exposed to occupational noise.

Tonal audiometry was carried out using a model OB-88 Madsen audiometer.

DPOAE were recorded through an otoacoustic emissions analyzer from Bio-logic Systems Corporation, Scout Sport (Distortion Product Otoacoustic Emissions Measurement System - version 1.54 program).

The technical criteria used to calculate the noise dose used in this study^12^ is in agreement with those from the Ministry of Employment and Labor^3^.

## RESULTS

Table 1 shows that 58.1% of the workers who worked in areas with environmental noise above the tolerance levels had audiometric test results within acceptable limits.

**Table 1:** Distribution of the individuals assessed through tonal audiometry as to occupational noise exposure and the audiometric test result, Capital Campus - USP, 2002.

Exposure to Occupational noise	Tonal audiometry within acceptable limits ** n % (IC*)	Altered tonal audiometry n % (IC*)	Total n %
Exposure within tolerance limits ***	142 80,2 (73,9-85,6)	35 19,8 (14,3-26,0)	177 100,0
Exposure above tolerance limits	50 58,1 (47,6-68,1)	36 41,9 (31,8-52,3)	86 100,0
Total	192 73,0 (67,4-78,1)	71 27,0 (21,8-32,5)	263 100,0

* 95% Confidence Interval.

** Acceptable tonal audiometry based on Ordinance # 19 – Ministry of Employment and Labor (BRAZIL, 1998).

*** Tolerance limits based on NR # 15 (BRAZIL, 1978).

DPOAE responses were considered for each individual in groups I and II in the frequencies of 3,000; 4,000 and 6,000Hz (Table 2), because it is in this frequency range that we have the earliest ONIHL alterations.

**Table 2:** Odds Ratio (OR) of Distortion Product Otoacoustic Emissions(DPOAE) response absence, in 3, 4 and 6kHZ, in the groups of those exposed and not exposed to occupational noise; Capital Campus - USP, 2002.

Groups	3, 4 e 6 KHz		OR	IC - 95%**
	DPOAE	DPOAE		
	Absent	Present		
Exposed to occupational noise* n=37	19	18	1	
Not exposed to occupational noise n=37	3	34	12,0	3,1 - 45,9

* Exposure based on NR # 15 (BRAZIL 1978) tolerance limits.

** 95% confidence interval.

An Odds Ratio of 12, as a risk estimate of having response absence in DPOAE due to occupational noise exposure, was statistically significant (CI 95% = 3.1-45.9).

The prevalence of no DPOAE response (Table 3) was greater for those workers with noise doses above 1.5 (77%) than for those with noise dose between 1 and 1.5 (37.5%).

**Table 3:** Number and percentage of results in the Distortion Product Otoacoustic Emissions (DPOAE) records in relation to the noise dose calculated for the USP Capital Campus workers exposed to occupational noise, 2002.

Noise Dose*	DPOAE		Total n %
	Present	Absent	
	n %	n %	
From 1 to 1.5	15 62,5	9 37,5	24 100,0
Greater than1.5	3 23,0	10 77,0	13 100,0
Total	18 48,6	19 51,4	37 100,0

* Noise Dose = C1 + C2 +. = Ci, where: T1 T2 Ti Ci is the total daily time, in minutes, in which the worker is exposed to sound pressure level corresponding to the iesima acoustic situation; Ti is the maximum daily exposed time, in minutes, allowable to the level corresponding to the iesima acoustic situation (FUNDACENTRO 1985). Fisher Exact Test, p = 0.038.

The association between the DPOAE recording results and the calculated noise dose was statistically significant (p = 0.038).

## DISCUSSION

The results of this investigation suggest that there is an association between no DPOAE responses for workers exposed to occupational noise when compared to those not exposed, exactly within that frequency range where the initial auditory lesions occur, as other studies show^7^, ^13^.

Oliveira et al.^14^ suggested the usefulness of evoked otoacoustic emissions, specially DPOAE, in the early identification of cochlear alterations that preceded the onset of ONIHL.

Fukuda^15^ stated that DPOAE are impaired in the high frequencies in individuals exposed to noise and according to the individual's hearing threshold, established by audiometry, as it increases, distortion product amplitudes reduce. DPOAE would aid in the diagnosis of ONIHL and it is important in its follow up.

The prevalence found (41.9%) for any tonal audiometry alteration, among those exposed to occupational noise, was similar to that of 40.4% found in the study by Corrêa Filho et al.^3^.

As to those individuals with audiometric results within acceptable limits, among the workers exposed to occupational noise, there was a greater prevalence than that found in other studies. Fiorini^9^ obtained 45.3% of individuals exposed to occupational noise with normal audiometric exams. Notwithstanding, the author used a more rigorous criteria to classify the tests as normal.

In the present study, the individuals classified as having altered tonal audiometry encompassed both ONIHL suggestive cases and those with other alterations.

The noise dose calculation was carried out considering the characteristics of the institution under study. Not being truly a company or factory, where production happens in a continuous fashion, there was the concern of evaluating the workers beyond their work environment, through sound pressure levels, the intermittent exposure of each individual in their workplace.

As we used the noise dose calculation method for the present investigation we had to consider a less accurate measure of exposure, in comparison to the dosimeter use. Notwithstanding, being that the sound pressure levels in the environments where each worker worked were known and data from the individual interview were used to assess the characteristics of each work done and analyze exposure intermittence, we considered such method adequate to the objectives of this paper.

The occupational noise exposure assessment through the dose analysis has been the subject of very few investigations; however, we noticed a trend in more recent studies in considering exposure intermittence^16^, ^17^, ^18^, ^19^.

Results suggest that higher exposure doses of occupational noise exposure may cause greater proportions of cochlear lesions detectable by DPOAE. These results corroborate the idea that DPOAE tests may be useful in identifying initial hearing alterations caused by noise, still undetected by tonal audiometry, as other studies have suggested^7^, ^13^, ^20^.

The possibility of hearing alterations early detection through DPOAE and being related to the work environment allows for individual and collective protection actions to benefit the workers exposed to different hearing risk factors. Such protection measures could be implemented or enhanced even before a hearing disorder would happen and cause symptoms or irreversible damage.

## CONCLUSION

There was a correlation between occupational noise exposure and lack of DPOAE responses, and there was also a relationship between exposure to higher doses of occupational noise and the findings of alterations in otoacoustic emissions.

The Odds Ratio result for absent responses in DPOAE was greater for workers exposed to occupational noise when we consider the frequencies of 3,000Hz, 4,000Hz and 6,000Hz altogether.

The use of DPOAE seem useful as a method to detect early physiopathological alterations caused by exposure to occupational noise, proving itself to be a promising ancillary tool in ONIHL diagnosis.
